# Targeting post-translational modifications to overcome glioblastoma therapy resistance: emerging strategies and natural compounds

**DOI:** 10.3389/fonc.2026.1984427

**Published:** 2026-10-15

**Authors:** Tetsade Piermartiri, Ana Carolini Adriana da Matta, João Victor Krüger Pinto, Carla Inês Tasca, Cláudia Beatriz Nedel

**Affiliations:** 1 Graduate Program in Biochemistry, Department of Biochemistry, Federal University of Santa Catarina, Florianópolis, Santa Catarina, Brazil; 2 Graduate Program in Cell and Developmental Biology, Department of Cell Biology, Embryology and Genetics, Federal University of Santa Catarina, Florianópolis, Santa Catarina, Brazil

**Keywords:** glioblastoma, glioblastoma stem cells, natural compounds, post-translational modifications, PTM crosstalk, temozolomide, therapy resistance

## Abstract

Post-translational modifications (PTMs) regulate multiple mechanisms associated with glioblastoma (GBM) therapy resistance, which remains a major challenge in GBM treatment. Some resistance mechanisms involve genetic heterogeneity, glioblastoma stem cells, the tumor microenvironment, autophagy, metabolic reprogramming, vascular adaptation, and altered transcriptional regulation. By regulating protein activity, stability, localization, interactions, and signaling, PTMs can influence several of these processes and affect treatment response. This review examines the roles of phosphorylation, ubiquitination and ubiquitin-like modifications, acetylation, methylation, and glycosylation in resistance to temozolomide, radiotherapy, and targeted therapies. We further discuss emerging PTMs, including lactylation, succinylation, and UFMylation, for which evidence linking individual modifications to therapeutic resistance ranges from direct resistance mechanisms to effects primarily associated with GBM biology. PTM crosstalk is also discussed in the context of DNA repair, redox homeostasis, metabolism, glioblastoma stem cell maintenance, and therapy response. Finally, we examine pharmacological targeting of PTM-regulating enzymes and pathways and the effects of natural bioactive compounds on PTM-dependent mechanisms.

## 1 Introduction

Malignant primary brain tumors are particularly difficult to treat because of their infiltrative growth and resistance to conventional therapies. Gliomas comprise a heterogeneous group of primary central nervous system (CNS) tumors, among which glioblastoma (GBM) is the most aggressive adult-type diffuse glioma. According to the fifth edition of the World Health Organization Classification of Tumors of the Central Nervous System (WHO CNS5), GBM is defined as an IDH-wildtype adult-type diffuse glioma and classified as CNS WHO grade 4 (1). GBM is characterized by extensive molecular heterogeneity and rapid progression (2). Despite advances in surgery, radiotherapy, and chemotherapy, therapeutic responsiveness remains limited, with a median overall survival of approximately 15 months (3, 4).

Drug resistance constitutes a major obstacle to effective GBM treatment, contributing to tumor recurrence, therapeutic failure, and poor clinical outcomes. Resistance emerges from both genetic evolution and non-genetic adaptation. Intratumoral genomic heterogeneity generates genetically distinct subclones, whereas transcriptional and phenotypic plasticity enable tumor cells to reversibly adapt to therapeutic pressure (5).

Resistance can be broadly classified as intrinsic or acquired. Intrinsic resistance arises from pre-existing genetic, molecular, cellular, or microenvironmental features that reduce treatment sensitivity. In contrast, acquired resistance emerges during or after therapeutic exposure through genetic selection and/or non-genetic responses. Such responses may include selection of resistant genetic variants, signaling changes and reduced intracellular drug accumulation, including upregulation of drug-efflux transporters (6).

Beyond genetic alterations, resistance can also involve changes at the protein level through post-translational modifications (PTMs). By regulating protein activity, stability, localization, and interactions, PTMs can influence signaling and transcriptional reprogramming, thereby contributing to therapeutic resistance.

PTMs expand proteomic diversity through the covalent addition or removal of chemical groups or protein modifiers at specific amino acid residues during or after translation. These modifications influence protein conformation, localization, stability, activity, and interaction networks. To date, hundreds of PTMs have been identified (7). PTMs are regulated by specific enzyme families, including kinases, phosphatases, acetyltransferases, deacetylases, ubiquitin ligases, deubiquitinases, and enzymes involved in Small Ubiquitin-like MOdifier (SUMO) conjugation and deSUMOylation. Dysregulation of these regulatory systems can alter signaling and protein homeostasis, contributing to tumor progression and therapeutic resistance (8, 9).

This review integrates evidence on classical and emerging PTMs implicated in the response to temozolomide (TMZ), radiotherapy, and targeted therapies in glioma, with particular emphasis on GBM. We examine how phosphorylation, ubiquitination and ubiquitin-like modifications, acetylation, methylation, glycosylation, and less-characterized PTMs can contribute to major resistance programs, including GBM stem cells (GSC) maintenance and plasticity, responses to the tumor microenvironment, immune evasion, autophagy, metabolic reprogramming, angiogenesis and vasculogenic mimicry, and deregulated transcription factor networks. Particular attention is given to the molecular mechanisms linking specific PTMs and their regulatory enzymes to therapeutic resistance, as well as to the functional consequences of PTM crosstalk. We further discuss pharmacological strategies targeting PTM-regulating pathways and examine the emerging role of natural bioactive compounds as modulators of PTM-dependent signaling and as potential adjunctive strategies for modulating therapeutic resistance.

## 2 Understanding tumor resistance

Tumor resistance remains a major limitation to treatment efficacy. Genetic mutations remain among the most extensively studied contributors to drug resistance. Alterations in key genes can induce conformational changes that reduce drug binding, drive constitutive activation of pro-survival or DNA repair pathways, or disable regulators that normally restrain proliferation. In GBM, the DNA damage response (DDR) and replication stress response pathways can further contribute to resistance by promoting efficient repair of therapy-induced lesions, stabilizing stalled replication forks, and preventing lethal genomic instability during chemotherapy and radiotherapy (10, 11).

However, GBM resistance also involves GSCs, the tumor microenvironment, autophagy, metabolic reprogramming, vascular adaptation, and altered transcriptional regulation, many of which are regulated or reinforced by PTMs and are briefly outlined below.

### 2.1 Glioblastoma stem cells

Cancer stem-like cells have been implicated in therapeutic resistance across multiple malignancies; in GBM, these populations are referred to as GSCs and represent stem-like cellular populations within the tumor that contributes to GBM evolution, malignant progression, and therapeutic resistance. In a genetically engineered mouse glioma model, a relatively quiescent Nestin-marked tumor-cell subset survived TMZ and repopulated the tumor, whereas selective ablation of this population impaired tumor growth and enhanced the effect of TMZ (12). Furthermore, the ability of GSCs and non-stem tumor cells to adapt to diverse microenvironments and withstand therapeutic stress is supported by their capacity to undergo reversible transitions between distinct cellular states. This process, termed phenotypic plasticity, is influenced by multiple factors, including cell-intrinsic pathways, microenvironmental cues, and the localization of GSCs within specialized hypoxic, invasive, or perivascular niches (3).

### 2.2 Tumor microenvironment

The tumor microenvironment (TME) consists of malignant cells, GSCs, immune cells, vasculature, stromal components, the extracellular matrix (ECM), and locally produced cytokines. In GBM, hypoxia, altered metabolite accumulation, and ECM remodeling create microenvironmental conditions that can support tumor-cell survival and reduce therapeutic efficacy. Rapid tumor expansion generates hypoxic niches where insufficient perfusion induces hypoxia-inducible factor 1 alpha (HIF-1α) stabilization, promoting glycolytic metabolism and other adaptive responses. Accumulation of metabolites such as lactate and CO_2_ contributes to extracellular acidification. Tumor-derived remodeling of the ECM produces a denser and stiffer matrix that enhances invasion while physically impeding chemotherapeutic drug penetration. Together, these microenvironmental alterations create a niche that supports tumor survival, plasticity, and resistance to therapy (13). Additionally, the interactions of GSCs with stromal components of the TME further reinforce these adaptive resistant phenotypes, facilitating survival, therapeutic evasion, and tumor regrowth (3).

### 2.3 Immune evasion

Immune evasion in GBM is influenced by myeloid populations within the TME. GBM-associated myeloid cells include resident microglia and recruited monocyte-derived macrophages, collectively contributing to the tumor-associated macrophage (TAM) population. Rather than existing as discrete antitumoral M1 or protumoral M2 phenotypes, TAMs occupy a spectrum of activation states, many of which acquire immunosuppressive and tumor-supportive functions in response to tumor-derived signals. Tumor-supportive TAM phenotypes can suppress cytotoxic T-cell activation, promote invasion, and enhance angiogenesis. TAMs secrete cytokines that can induce stem-like properties in tumor cells, reinforcing tumorigenesis, and contributing to therapeutic resistance (13). In primary GBM, myeloid cells expressing *HMOX1*, the gene encoding heme oxygenase-1 (HO-1), spatially associate with dysfunctional T cells and promote interleukin-10 (IL-10)-dependent immunosuppression, a mechanism functionally reproduced in a human neocortical GBM model (14).

### 2.4 Autophagy

Autophagy is a conserved catabolic process in which cytoplasmic material is sequestered into double-membrane autophagosomes that subsequently fuse with lysosomes, where the cargo is degraded and its components can be recycled for bioenergetic and biosynthetic processes. Autophagy has context-dependent roles in cancer and can promote either tumor-cell survival or cell death depending on the cellular context. In GBM, autophagy can support adaptation to therapeutic stress by maintaining cellular homeostasis and recycling damaged cellular components (4). Temozolomide can induce cytoprotective autophagy in GBM cells, potentially limiting treatment-induced cell death (15). Accordingly, knockdown of autophagy-related 9B (ATG9B) suppresses TMZ-induced autophagy and increases TMZ sensitivity in GBM cells (16).

### 2.5 Metabolic reprogramming in GBM

Metabolic reprogramming is a hallmark of GBM and can contribute to therapeutic resistance. GBM cells frequently exhibit enhanced aerobic glycolysis, consistent with the Warburg effect, with increased glucose utilization and lactate production even in the presence of oxygen. Combined inhibition of glycolysis with dichloroacetate and fatty-acid oxidation with ranolazine reduced GBM clonogenic growth and increased survival in orthotopic syngeneic models (17).

Beyond glycolysis, GBM exhibits extensive remodeling of lipid metabolism. TMZ-resistant GBM cells showed increased lipid synthesis and sterol response element-binding protein (SREBP) signaling, and inhibition of SREBP with fatostatin preferentially impaired resistant cells, an effect also observed in patient-derived TMZ-resistant cells (18, 19).

### 2.6 Angiogenesis and vasculogenic mimicry

Angiogenesis, the formation of new blood vessels, sustains the metabolic demands of GBM. However, when angiogenesis is blocked, such as with anti-vascular endothelial growth factor (VEGF) therapies, GBM can shift to alternative neovascular mechanisms to maintain tumor perfusion and contribute to therapeutic resistance. Reduced perfusion caused by anti-VEGF drugs can exacerbate hypoxia, which stabilizes HIF-1α and HIF-2α. These transcription factors (TFs) upregulate multiple pro-angiogenic genes and drive compensatory responses. As a result, GBM can switch to alternative modes of vascularization, including vessel co-option (tumor cells grow along and use existing blood vessels), vasculogenic mimicry (VM) (tumor cells form vessel-like channels without endothelial cells), and transdifferentiation of GSCs into cells with vascular-like properties. These mechanisms may help sustain tumor perfusion during VEGF inhibition and have been proposed to contribute to the limited clinical benefit of anti-angiogenic therapy, which can improve progression-free survival but not overall survival (20). Recent work identified a therapy-induced, partially reversible vessel-co-opting GBM state (VC-Resist), directly linking vascular adaptation and cellular plasticity to chemoradiation resistance (21).

### 2.7 Deregulation of transcription factors

Numerous TFs are aberrantly expressed or activated in gliomas and are associated with malignant features, including uncontrolled proliferation, invasion, stemness, and immune evasion. Among them, signal transducer and activator of transcription 3 (STAT3) is one of the most extensively implicated. STAT3 is activated primarily through phosphorylation at tyrosine 705 (Y705), typically mediated by Janus kinases (JAKs) or alternative cytoplasmic kinases, a modification that promotes its dimerization, nuclear translocation, and transcriptional activity. Constitutive STAT3 activation in GBM promotes tumor cell proliferation and migration, sustains GSC properties, enhances angiogenesis and contributes to immunosuppressive remodeling of the TME, thereby supporting resistance to therapy (22).

## 3 PTMs and their role in tumor resistance

PTMs expand proteomic diversity and regulate protein responses to cellular and environmental stimuli. Many PTMs regulate protein function through enzymes that install, remove, or recognize specific modifications. Dysregulation of these networks contributes to various diseases, including cancer, where PTMs influence signaling pathways, metabolic reprogramming, and immune regulation associated with tumor progression and resistance to therapy. **Figure 1** provides an illustration of the major PTMs discussed in this review and the principal resistance-related mechanisms through which they can influence GBM therapeutic response.

**Figure 1:**
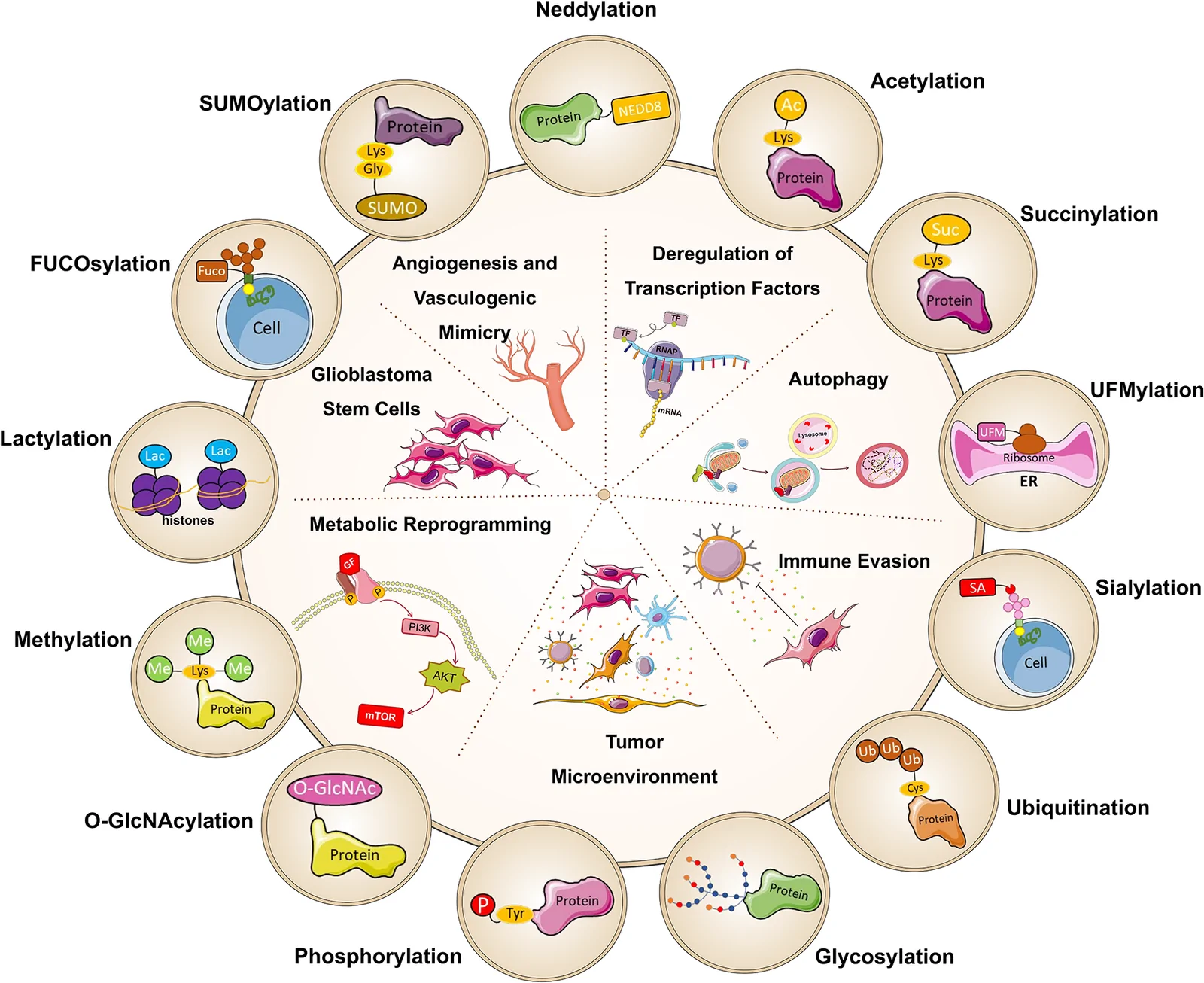
Post-translational modification involved in glioblastoma therapy resistance. Major PTMs and the GBM-associated processes through which they may influence therapeutic response are schematically represented.

### 3.1 Phosphorylation

Phosphorylation, the reversible addition of a phosphate group to serine, threonine, or tyrosine residues, is one of the most ubiquitous and extensively characterized PTMs. Phosphorylation is catalyzed by a large and diverse family of protein kinases, whereas dephosphorylation is mediated by phosphatases. At the plasma membrane, receptor tyrosine kinases (RTKs) such as epidermal growth factor receptor (EGFR), vascular endothelial growth factor receptor (VEGFR), and fibroblast growth factor receptor (FGFR) initiate signaling cascades (9).

The addition of a negatively charged phosphate group can induce conformational changes within a protein, modulating its enzymatic activity, subcellular localization, stability, and interactions with binding partners. This dynamic remodeling of phosphorylation-dependent signaling enables cells to integrate environmental cues and stress responses rapidly. Phosphatases also display significant substrate selectivity and contribute actively to both oncogenic and tumor-suppressive signaling networks (8, 9).

In GBM, aerobic glycolysis is a prominent metabolic feature, but glutamine metabolism also contributes to metabolic reprogramming. During glutaminolysis, glutamine is first converted to glutamate by glutaminase, whereas glutamate dehydrogenase 1 (GDH1) catalyzes the subsequent conversion of glutamate to α-ketoglutarate (α-KG), which can enter the tricarboxylic acid (TCA) cycle. This metabolic pathway can be stimulated by EGFR signaling, which is genetically altered in approximately 50% of GBMs. EGFR signaling through mitogen-activated protein kinase kinase (MEK)/extracellular signal-regulated kinase (ERK) promotes phosphorylation of ETS-like protein 1 (ELK1), increasing *GDH1* transcription and promoting glutamine metabolism (23).

In turn, increased GDH1 activity and α-KG production can reinforce intracellular signaling and glycolytic metabolism in GBM. α-KG increases pyruvate dehydrogenase kinase 1 (PDPK1) transcription through KDM6A-dependent reduction of the repressive histone H3 lysine 27 trimethylation (H3K27me3) mark, thereby amplifying EGFR-mediated phosphoinositide 3-kinase (PI3K)/protein kinase B (AKT)/mechanistic target of rapamycin (mTOR) signaling. GDH1-derived α-KG also promotes hexokinase 2 (HK2) expression and glycolytic flux through KDM6A-mediated H3K27 demethylation. Thus, increased GDH1 activity and α-KG production can connect glutamine metabolism with PI3K/AKT/mTOR signaling and glycolysis (24). Notably, metabolic adaptations involving both glycolysis and glutamine metabolism have been associated with therapeutic resistance in GBM (25).

A related metabolic vulnerability has been demonstrated in acquired TMZ-resistant EGFR variant III (EGFRvIII)-positive GBM cells, in which inhibition of pyruvate dehydrogenase kinase 1 (PDK1) with dichloroacetate (DCA) reversed the Warburg phenotype, increased oxidative mitochondrial metabolism, and induced apoptosis (26), linking kinase-regulated metabolism to metabolic changes associated with TMZ resistance.

Phosphorylation-associated PI3K/AKT signaling can also support adaptive resistance to TMZ, although these studies do not establish individual phosphorylation events as the initiating cause of resistance. In paired GBM samples collected before and after treatment, TMZ exposure was associated with enrichment of resistant aldehyde dehydrogenase 1 family member A1 (ALDH1A1)^+^/phosphorylated AKT (pAKT)^+^ subclones. Sequential treatment with TMZ followed by AKT inhibition reduced the expansion of these resistant populations in patient-derived models, indicating that AKT activation can support the emergence of TMZ resistance (27). The PI3K inhibitor XH30 reduced AKT phosphorylation and downstream PI3K signaling and increased TMZ sensitivity in resistant GBM models. This effect was also associated with repression of glioma-associated oncogene family zinc finger 1 (GLI1), a transcription factor of the Hedgehog pathway (28). Similarly, the irreversible pan-PI3K inhibitor PX-866 suppressed PI3K/AKT signaling and TMZ-induced cytoprotective autophagy, increasing apoptosis and reducing clonogenic survival when administered after TMZ (15).

Phosphorylation-dependent signaling can also contribute to resistance to targeted therapies. When a dominant kinase pathway is inhibited, GBM cells can redirect signaling through alternative RTKs or downstream kinase networks. For example, EGFR inhibition can de-repress platelet-derived growth factor receptor β (PDGFRβ) expression and shift tumor-cell dependence toward PDGFRβ signaling, providing a mechanism of acquired resistance to EGFR inhibitors (29). Activation of insulin receptor (InsR)/insulin-like growth factor 1 receptor (IGF1R) signaling can similarly maintain survival during EGFR inhibition (30), whereas EGFR blockade can trigger increased tumor necrosis factor (TNF) signaling, followed by activation of c-Jun N-terminal kinase (JNK), AXL receptor tyrosine kinase, and ERK, thereby promoting primary resistance to EGFR inhibition. Single-cell phosphoproteomic analysis of a patient-derived GBM model showed that mTOR inhibition altered signaling coordination within 2.5 days of treatment, before overt resistance developed (31). Combination therapies selected from these early adaptive changes achieved sustained tumor suppression *in vivo* (32).

These mechanisms also provide a rationale for pharmacologically targeting phosphorylation-dependent signaling, although the response depends on the molecular context and treatment strategy. In the EGFRvIII-positive D317 GSC model, osimertinib inhibited constitutive EGFRvIII kinase activity and downstream signaling and suppressed tumor growth in heterotopic and orthotopic xenografts (33).

Phosphorylation-dependent signaling has also been associated with resistance to anti-angiogenic therapy. In GBM specimens obtained after the development of resistance to bevacizumab, increased c-Met phosphorylation was accompanied by increased phosphorylation of its downstream effectors focal adhesion kinase (FAK) and STAT3. These changes were associated with a more invasive phenotype, supporting activation of the c-Met signaling as a mechanism of adaptation to bevacizumab treatment (34).

Pharmacological inhibition of kinase signaling can also enhance TMZ response without establishing phosphorylation as the underlying resistance mechanism. The AKT inhibitor perifosine synergized with TMZ in U87MG and U251 glioblastoma cells, decreased breast cancer type 1 susceptibility protein (BRCA1) expression and DNA-repair activity, increased DNA damage and apoptosis, and enhanced tumor suppression in xenografts (35). Treatment sequence may be particularly important when combining kinase inhibitors with TMZ. EGFR inhibition was shown to reduce *O*6-methylguanine-DNA methyltransferase (MGMT) expression through an activator protein 1 (AP-1)/microRNA-616 (miR-616)-dependent mechanism. Concomitant exposure to TMZ counteracted this effect, whereas EGFR tyrosine kinase inhibitor (EGFR-TKI) pretreatment followed by TMZ maintained MGMT downregulation and increased TMZ sensitivity in experimental GBM models. Analysis of post-treatment tissues from two clinical trials further supported the regulation of MGMT by EGFR inhibition, providing a mechanistic rationale for administering EGFR inhibitors before TMZ, particularly in MGMT-unmethylated GBM (36).

Phosphorylation-dependent responses are also observed following radiotherapy. Phosphoproteomic profiling of U251 cells exposed to X-ray irradiation revealed extensive remodeling of DDR-associated phosphoproteins, including BRCA1, mediator of DNA damage checkpoint 1 (MDC1), histone H2A.X (H2AX), and tumor protein p53-binding protein 1 (TP53BP1), although the causal contribution of these individual phosphorylation events to radioresistance remains to be established (37).

Finally, phosphorylation can interact with other PTMs, including ubiquitination; these mechanisms are discussed in the dedicated PTM crosstalk section.

### 3.2 Ubiquitination

Ubiquitination is a PTM in which ubiquitin, a small protein of 76 amino acids, is covalently attached most commonly to lysine residues of a substrate protein. Ubiquitin attachment occurs through a three-enzyme cascade that involves an E1 ubiquitin-activating enzyme, an E2 ubiquitin-conjugating enzyme, and an E3 ubiquitin ligase, whereas ubiquitin modifications can be reversed by deubiquitinases (DUBs) (7).

The functional outcome of ubiquitination on the modified protein depends on the type of ubiquitin chain that is formed. Ubiquitin contains eight possible linkage sites, including seven lysine residues (K6, K11, K27, K29, K33, K48, and K63) and one N-terminal methionine residue (M1). K48-linked polyubiquitination generally marks proteins for degradation by the proteasome. In contrast, other chain types such as K63-linked polyubiquitination participate in non-proteolytic processes, including the regulation of DNA repair, signal transduction, endocytic trafficking, and autophagic degradation (38). In GSCs, ubiquitin-specific peptidase 14 (USP14)-mediated deubiquitination of AlkB homolog 5 (ALKBH5) limits its K48-linked ubiquitination and proteasomal degradation, thereby stabilizing ALKBH5 and supporting homologous-recombination repair and radioresistance. Pharmacological inhibition of USP14 with IU1 disrupted this pathway and enhanced the response of GSC-derived brain tumor xenografts to irradiation (39).

Depending on the substrate, ubiquitination and deubiquitination can either promote or limit therapeutic resistance by controlling the stability of proteins involved in survival, metabolism, and DNA repair. In GBM, this balance is relevant to TMZ resistance because ubiquitination regulates the stability of proteins involved in alkylation-damage repair. The deubiquitinase ubiquitin-specific peptidase 7 (USP7) removes K48-linked ubiquitin chains from MGMT, preventing its proteasomal degradation and maintaining MGMT-mediated repair. Accordingly, genetic or pharmacological inhibition of USP7 promotes MGMT degradation and increases TMZ sensitivity (40). Conversely, in MGMT-deficient GBM, the E3 ligase LIM domain only 7 (LMO7) promotes K48-linked polyubiquitination and proteasomal degradation of ALKBH2, another enzyme involved in alkylation-damage repair. Loss of ALKBH2 increases TMZ sensitivity, indicating that ubiquitin-dependent regulation of DNA repair can influence TMZ response even in the absence of MGMT (41).

Ubiquitination can also promote TMZ resistance through non-proteolytic DNA-damage tolerance. In MGMT-deficient GBM cells, mismatch repair processing of TMZ-induced O6-methylguanine lesions generates single-stranded DNA (ssDNA) gaps that recruit the E3 ubiquitin ligase RAD18, leading to monoubiquitination of proliferating cell nuclear antigen (PCNA) and recruitment of the Y-family DNA polymerase kappa (Polκ) for post-replicative repair. RAD18 depletion increases TMZ sensitivity in established and patient-derived GBM models (42).

Ubiquitination can also influence TMZ response by regulating tumor metabolism. In glioma models, the E3 ubiquitin ligase C-terminus of Hsc70-interacting protein (CHIP) promotes K48-linked ubiquitination of branched-chain amino acid transaminase 1 (BCAT1), with K360 identified as a major ubiquitination site controlling BCAT1 stability. CHIP-mediated BCAT1 degradation reduces glutathione synthesis and increases oxidative stress, thereby enhancing TMZ sensitivity. Importantly, expression of the ubiquitination-resistant BCAT1-K360R mutant counteracted CHIP-mediated TMZ sensitization, supporting a functional role for BCAT1 ubiquitination in determining TMZ response (43).

More recently, ubiquitin C-terminal hydrolase L1 (UCHL1) was identified as a mediator of TMZ resistance in GBM. UCHL1 removes K27-linked polyubiquitin chains from keratin 8 (KRT8), preventing its proteasomal degradation and maintaining KRT8 stability. Genetic depletion or pharmacological inhibition of UCHL1 increased TMZ sensitivity, whereas KRT8 overexpression partially restored resistance, supporting the UCHL1–KRT8 axis as a mechanism of chemoresistance. Notably, UCHL1 depletion also increased the sensitivity of GBM cells to lomustine (CCNU), a clinically used alkylating agent, although the specific mechanism underlying this effect was not further characterized (44).

Collectively, these studies show that ubiquitination regulates both protein degradation and non-proteolytic signaling processes, with distinct effects on therapeutic response depending on the target involved.

### 3.3 SUMOylation, neddylation, ISGylation

SUMOylation, neddylation, and ISGylation are PTMs mediated by ubiquitin-like proteins (UBLs) that share structural features and conjugation mechanisms with ubiquitin but regulate distinct cellular processes. The major modifiers discussed here are SUMO, Neural precursor cell Expressed Developmentally Downregulated 8 (NEDD8), and Interferon-Stimulated Gene 15 (ISG15).

SUMOylation is a reversible modification that involves the covalent attachment of SUMO proteins to lysine residues of target proteins, thereby modulating their stability, subcellular localization, and transcriptional activity. SUMO proteins are approximately 100 amino acids in length, and the major human paralogs include SUMO1, SUMO2, SUMO3, and SUMO4; because of their high sequence similarity, SUMO2 and SUMO3 are commonly referred to as SUMO2/3. In glioma and GBM models, dysregulation of SUMOylation has been associated with stemness, vascular remodeling and responses to genotoxic stress. SUMOylation occurs through an enzymatic cascade similar to ubiquitination and requires sequential action of E1 activating enzymes, E2 conjugating enzymes, and E3 ligases. SUMO is activated by the E1 enzyme complex, transferred to the E2 ubiquitin-conjugating enzyme 9 (Ubc9), and finally attached to a lysine residue on the substrate, often facilitated by E3 ligases such as members of the protein inhibitor of activated STAT (PIAS) family. The modification is reversible, and deSUMOylation is mediated by sentrin-specific proteases (SENPs), which remove SUMO from modified substrates (45).

SUMOylation can support the GSC maintenance. GSCs exhibit increased SUMO1 conjugation, and the peptidyl-prolyl cis-trans isomerase NIMA-interacting 1 (Pin1) promotes SUMOylation of promyelocytic leukemia protein (PML), facilitating its interaction with and stabilization of the transcription factor c-Myc and thereby supporting GSC self-renewal and tumorigenic capacity (46).

SUMOylation also contributes to GBM responses to hypoxia. Under hypoxia, increased Ubc9 expression enhances SUMOylation of heterogeneous nuclear ribonucleoprotein A2/B1 (hnRNP A2/B1), linking SUMOylation to vascular remodeling. Pharmacological inhibition of SUMOylation with TAK-981 reduced hnRNP A2/B1 SUMOylation, disrupted microRNA-204-3p (miR-204-3p) exosomal sorting, and inhibited GBM growth and angiogenesis. However, because TAK-981 has limited blood–brain barrier permeability, its *in vivo* activity was evaluated in a subcutaneous rather than intracranial GBM model (47).

A direct link between SUMOylation and resistance to genotoxic therapy has been demonstrated for the oligodentrocyte transcription factor 2 (Olig2). SUMO1 conjugation at K27, K76, and K112 enables Olig2 to suppress p53-dependent cell-cycle arrest and apoptosis following DNA damage. Loss of these SUMOylation sites reduces the protective effect of Olig2 and increases TMZ sensitivity, demonstrating that SUMOylation of a specific transcription factor can directly influence chemotherapy response (48).

Neddylation is a reversible UBL modification involving the covalent conjugation of NEDD8 to target proteins through an enzymatic cascade analogous to ubiquitination. NEDD8 is activated by the NEDD8-activating enzyme (NAE), composed of NAE1 and the catalytic ubiquitin-associated domain (UBA3), in an ATP-dependent manner. The activated modifier is transferred to one of two E2 conjugating enzymes, ubiquitin conjugating enzyme E2 F and M (UBE2F, UBE2M), and subsequently conjugated to a lysine residue on the target protein, with the assistance of E3 ligases such as gene finger domain (RING)-box protein 1 and 2 (RBX1, RBX2). Like SUMOylation, the process is reversible, and NEDD8 can be removed by deneddylating machinery, including the constitutive photomorphogenesis 9 (COP9) signalosome and its catalytic COP9 signalosome homolog subunit 5 (CSN5). Cullins are among the best-characterized NEDD8 substrates, and their neddylation activates cullin-RING ubiquitin ligases, thereby linking neddylation to the control of protein stability and cellular signaling (49).

Neddylation inhibition can also affect GSC maintenance. Treatment with the NAE inhibitor MLN4924 inhibited the growth of patient-derived GSC cultures, although sensitivity varied considerably among models. MLN4924-sensitive GSCs showed enrichment of ERK/AKT signaling, and treatment reduced ERK and AKT phosphorylation (50). Neddylation inhibition can also affect chemotherapy response. In TMZ-resistant GBM cell lines, combination treatment with MLN4924 and TMZ reduced cell viability and sensitized resistant cells to TMZ compared with TMZ treatment alone (51). However, the response to NAE inhibition is not uniform across GBM models. Loss of functional phosphatase and tensin homolog (PTEN) is associated with resistance to multiple NAE inhibitors, whereas sensitive models show higher baseline S-phase populations and, following MLN4924 treatment, greater DNA re-replication and DNA-damage responses (52).

ISGylation is a ubiquitin-like modification in which ISG15 is covalently attached to target proteins through an enzymatic cascade involving the E1-activating enzyme UBA7, the E2-conjugating enzyme UBCH8, and the E3 ligase HERC5. The modification is reversible, and removal of ISG15 is carried out by the deISGylating enzyme USP18. Unlike K48-linked polyubiquitination, ISGylation does not primarily function as a proteasomal degradation signal and can instead modulate substrate activity, stability, or interactions. Its effects in cancer are highly context dependent and can influence both tumor-cell signaling and immune responses (53). Evidence in glioma suggests that ISGylation can contribute to the maintenance of stem-like properties. ISG15-mediated ISGylation of the octamer-binding transcription factor 4 (Oct4) at K284 increases Oct4 stability and supports glioma cell stemness and tumor-initiating capacity (54). In GBM, increased levels of ISG15 and protein ISGylation have also been reported (55). However, direct evidence linking covalent ISGylation itself to TMZ or radiotherapy resistance in GBM remains limited.

### 3.4 Acetylation

Protein acetylation and methylation are PTMs that can contribute to epigenetic regulation when they occur on histones and influence chromatin structure and gene expression. Epigenetic mechanisms that directly modify DNA are beyond the scope of this review and have been extensively reviewed elsewhere (3, 56).

Acetylation involves the transfer of an acetyl group from acetyl-CoA to lysine side chains, where it can occur enzymatically or non-enzymatically. Enzymatic lysine acetylation is catalyzed by lysine acetyltransferases (KATs; historically termed histone acetyltransferases, HATs) and reversed by histone deacetylases (HDACs) (9). Histone acetylation is a key regulator of chromatin structure and gene expression, while numerous non-histone proteins are also acetylated, with important effects on protein stability and function. Moreover, acetylation can influence protein–protein interactions, subcellular localization, and the activity of mitochondrial proteins that regulate metabolic pathways (57).

Acetylation-dependent mechanisms can regulate GSC maintenance and plasticity. A recently described metabolic–epigenetic mechanism links mitochondrial metabolism directly to histone acetylation. Mitochondrial calcium uniporter (MCU)-driven mitochondrial Ca²^+^ uptake increases acetyl-CoA availability and histone H3K27 acetylation at the TRIB3 locus, sustaining TRIB3 expression, GSC self-renewal, and tumor-initiating capacity (58).

Non-histone acetylation also contributes to GSC resistance. Acetylation of Annexin A2 (ANXA2) at lysine 10 (K10) enhances its interaction with ubiquitin-specific peptidase 4 (USP4) and participates in an ANXA2 signaling network that supports GSC maintenance and radioresistance (59).

Acetylation also contributes to radiation-induced GSC plasticity. Following irradiation, p300 activity increases H3K27 acetylation and chromatin accessibility at vascular genes and promotes the conversion of GSCs toward vascular-like phenotypes. Pharmacological inhibition of p300 reversed these radiation-induced changes and reduced post-treatment tumor growth, linking histone acetylation to radiation-induced tumor recurrence (60). The p300 pathway also contributes to TMZ resistance through DNA-damage repair. The retinoblastoma-binding protein 4 (RBBP4)/p300 complex maintains the expression of homologous-recombination genes, including radiation sensitive protein 51 (RAD51), through H3K27 acetylation. Pharmacological inhibition of RBBP4/p300-HAT activity with CCS1477 or NEO2734 reduced H3K27ac and homologous-recombination activity, delayed repair of TMZ-induced DNA damage, and increased TMZ sensitivity in patient-derived xenograft (PDX)-derived GBM cells; in orthotopic PDX models, combination with TMZ provided a modest additional survival benefit (61).

HDAC-associated transcriptional regulation can also maintain TMZ resistance. In TMZ-resistant patient-derived GBM cells, HDAC1 cooperates with SRY-box transcription factor 2 (SOX2) at the PDGFB super-enhancer, sustaining platelet-derived growth factor subunit B (PDGFB) transcription and downstream mitogen-activated protein kinase (MAPK)/PI3K signaling. Disruption of this regulatory complex increases TMZ-induced DNA damage and apoptosis, demonstrating that HDAC1-dependent transcriptional regulation contributes to chemoresistance (62).

Despite the preclinical rationale for targeting acetylation-regulating enzymes, clinical translation of HDAC inhibition has been limited. In a phase I/II trial in newly diagnosed GBM, vorinostat combined with TMZ and radiotherapy was tolerable but did not meet its prespecified efficacy endpoint (63).

### 3.5 Methylation

Protein methylation commonly occurs on lysine and arginine residues. Lysine methylation is catalyzed by lysine methyltransferases (KMTs), which use *S*-adenosylmethionine (SAM) as the methyl donor to generate mono-, di-, or tri-methylated lysine (Kme1, Kme2, Kme3), whereas lysine demethylases (KDMs) remove these marks. Depending on the specific lysine residue and the degree of methylation, lysine methylation regulates chromatin structure, transcriptional activity, protein stability, and diverse signaling pathways (56). Here, we focus on protein methylation, including histone modifications, whereas mechanisms involving direct methylation of DNA are beyond the scope of this review.

For instance, the suppressor of variegation 3–9 homolog 1 (SUV39H1), a histone methyltransferase responsible for histone H3K9 trimethylation (H3K9me3), is upregulated in GBM and preferentially expressed in GSCs. Genetic depletion or pharmacological inhibition of SUV39H1 reduced GSC proliferation and stemness, while treatment with chaetocin sensitized GSCs to TMZ, supporting a role for this histone methyltransferase in GSC maintenance and chemotherapy response (64). Moreover, lysine methylation of non-histone proteins can also contribute to therapeutic resistance. In GBM cells with EGFR activation, AKT phosphorylates the lysine methyltransferase enhancer of zeste homolog 2 (EZH2) at S21, promoting its interaction with the osmoprotective nuclear factor of activated T cells 5 (NFAT5) and subsequent methylation of NFAT5 at K668. NFAT5 methylation promotes its stabilization and nuclear accumulation, increasing MGMT transcription and reducing TMZ efficacy (65).

On the other hand, lysine demethylase 5B (KDM5B) identifies a pre-existing slow-cycling cell population associated with subsequent TMZ resistance. KDM5B is preferentially active in rare stem-like ALDH1A1^+^ populations, and its expression increases following TMZ exposure. Treatment-naive KDM5B-high cells behave as slow-cycling persisters and show increased AKT phosphorylation, whereas KDM5B depletion increases PTEN expression, reduces AKT activation, and enhances TMZ sensitivity. Clonal tracing further showed that high KDM5B levels before treatment predict subsequent subclonal expansion during TMZ exposure, linking KDM5B activity to the early development of chemoresistance (66).

Lysine demethylase 5A (KDM5A) has also been implicated in acquired TMZ resistance. KDM5A expression is increased in TMZ-resistant GBM cells, while KDM5A overexpression promotes TMZ resistance and its inactivation restores TMZ sensitivity (67). Moreover, lysine demethylase 4C (KDM4C) contributes to acquired TMZ resistance by upregulating E2F transcription factor 6 (E2F6). Pharmacological inhibition of KDM4C with SD70 reduces proliferation and stem-like properties of TMZ-resistant cells and synergizes with TMZ, restoring TMZ sensitivity (68).

Arginine mono-, di-, or tri-methylation can also contribute directly to TMZ resistance through DNA-repair regulation. Protein arginine methyltransferase 5 (PRMT5), which catalyzes symmetric arginine dimethylation, supports TMZ resistance in GSCs by maintaining Fanconi anemia (FA)-dependent homologous-recombination repair. Genetic depletion or pharmacological inhibition of PRMT5 with LLY-283 suppresses this repair program, increases TMZ-induced DNA damage, and enhances the response to TMZ. The combination also reduced intracranial tumor growth and prolonged survival in GSC-derived xenograft models (69). PRMT5-dependent methylation can also support resistance to targeted therapy. During mTOR inhibition, PRMT5-mediated symmetric dimethylation of heterogeneous nuclear ribonucleoprotein A1 (hnRNP A1) at R218 and R225 maintains internal ribosome entry site (IRES)-dependent translation of cyclin D1 and c-Myc, allowing protein synthesis to persist despite mTOR blockade and contributing to resistance to mTOR inhibition (70).

Clinical translation of PRMT5 inhibition using the 5′-deoxy-5′-methylthioadenosine (MTA)-cooperative PRMT5 inhibitor BMS-986504 (MRTX1719) is currently being evaluated in a phase 0/1 trial in patients with recurrent methyladenosine phosphorylase (MTAP)-deleted GBM (https://clinicaltrials.gov/study/NCT06883747), but clinical efficacy has not yet been reported.

Additionally, PRMT5 activity has also been linked to adaptive resistance to MEK inhibition. In patient-derived GBM neurospheres, PRMT5 depletion enhanced the response to the MEK1/2 inhibitor trametinib and reduced treatment-induced AKT and receptor tyrosine-protein kinase erbB-3 (ERBB3) escape signaling; combined PRMT5 depletion and trametinib also prolonged survival in orthotopic xenograft models (71).

### 3.6 Glycosylation

Protein glycosylation comprises several co- and post-translational processes in which carbohydrate moieties are covalently attached to proteins. In the secretory pathway, glycosylation is broadly classified as *N*-linked or *O*-linked based on the amino acid to which the glycan is attached. Additional modifications such as sialylation and fucosylation can occur on both *N*-linked and *O*-linked glycans. Glycosyltransferases and glycosidases coordinate these reactions and altered glycosylation patterns have been associated with glioma progression, invasion, and therapeutic resistance (72, 73).

Recent evidence directly links altered sialylation to GSC radioresistance. N-acylneuraminate-9-phosphatase (NANP)-dependent regulation of tumor necrosis factor receptor 1 (TNFR1) sialylation sustains nuclear factor kappa B (NF-κB) signaling and the mesenchymal state of GSCs, whereas NANP suppression reduces TNFR1 sialylation, impairs DNA-damage repair, and increases radiosensitivity. This effect was also observed in intracranial GSC-derived tumors treated with fractionated radiotherapy (74).

GBM-associated sialoglycans can also contribute to immune evasion through engagement of sialic acid-binding immunoglobulin-like lectin (Siglec) receptors on myeloid cells. In preclinical GBM models, disruption of the Siglec–sialic acid axis enhanced myeloid-cell phagocytosis and antitumor immune responses and prolonged survival (75).

Fucosylation provides a direct link between glycosylation and TMZ response. Fucosyltransferase 8 (FUT8) alters the core fucosylation of hepatocyte growth factor receptor (MET) and EGFR, modulating hepatocyte growth factor (HGF)-induced MET signaling and EGFR transactivation; FUT8 suppression or treatment with the fucosylation inhibitor 2F-peracetyl-fucose increases TMZ sensitivity in GBM cells (76).

*O*-linked β-N-acetylglucosamine (*O*-GlcNAc) modification, or *O*-GlcNAcylation, is a distinct and dynamic intracellular modification in which *O*-GlcNAc transferase (OGT) adds *O*-GlcNAc to serine or threonine residues and O-GlcNAcase (OGA) removes it. In GBM, *O*-GlcNAcylation can support TMZ resistance through its effects on cellular survival and autophagy. Pharmacological inhibition of OGT with OSMI-1 reduces *O*-GlcNAcylation and autophagy and enhances TMZ-induced cell death, supporting *O*-GlcNAcylation as a mediator of chemoresistance (77).

More recently, metabolic context was shown to influence this vulnerability. In patient-derived GBM cells, hyperglycemia was associated with increased expression of hexosamine biosynthetic pathway enzymes and *O*-GlcNAcylation machinery. Inhibition of *O*-GlcNAcylation reduced viability and enhanced TMZ sensitivity, with a greater effect in cells derived from hyperglycemic patients (78).

The dependence of *O*-GlcNAc vulnerability on metabolic context further highlights the importance of considering tumor metabolism when developing targeted strategies for GBM treatment.

## 4 Emerging post-translational modifications in glioblastoma therapy resistance

Advances in proteomics, high-resolution mass spectrometry, and metabolically sensitive labeling strategies have expanded the catalog of known PTMs. Beyond the classical PTMs discussed above, several less-characterized modifications are emerging as potential regulators of therapeutic responses in glioma and GBM. For several emerging PTMs, evidence remains limited or primarily related to tumor biology; however, recent studies have established direct roles for selected modifications in GBM therapeutic resistance (7, 79).

Lactate, generated from pyruvate during glycolysis, functions not only as a metabolic by-product and energy substrate but also as a precursor for lysine lactylation, an emerging PTM implicated in diverse pathophysiological processes and therapeutic resistance. L-lactate can contribute to lysine lactylation through lactyl-CoA, while methylglyoxal can be converted to lactoylglutathione, which also participates in histone lysine lactylation (80). In GSCs with high expression of aldehyde dehydrogenase 1 family member A3 (ALDH1A3), metabolic reprogramming promotes X-ray repair cross-complementing protein 1 (XRCC1) lactylation at K247, enhancing its nuclear localization and DNA-repair activity and thereby supporting resistance to TMZ and radiotherapy (81). Histone lactylation provides a complementary mechanism of chemoresistance. H3K9 lactylation is increased in recurrent GBM and TMZ-resistant cells and promotes RNA-binding protein LUC7-like 2 (*LUC7L2*) transcription, leading to MutL homolog 1 (*MLH1*) intron 7 retention, reduced MLH1 expression, impaired mismatch repair, and TMZ resistance (82).

Succinylation is a lysine acylation PTM in which a succinyl group (−CO−CH_2_−CH_2_−CO_2_H) is covalently attached to the ϵ-amino group of lysine residues, using succinyl-CoA as the donor. Because succinyl-CoA is a metabolic intermediate closely linked to the TCA cycle, lysine succinylation provides a direct connection between cellular metabolism and the regulation of protein structure and function (83). In GBM, succinylation of phosphoglycerate kinase 1 (PGK1) at K191/K192 reduces its ubiquitin–proteasome-dependent degradation, stabilizing PGK1 and promoting aerobic glycolysis. Although this mechanism contributes to metabolic reprogramming and tumor growth, a direct role in resistance to standard GBM therapy has not yet been demonstrated (84).

UFMylation is an emerging ubiquitin-like modification in which ubiquitin-fold modifier 1 (UFM1) is conjugated to target proteins through an enzymatic cascade involving the E1 enzyme UBA5, the E2 ubiquitin-fold modifier conjugating enzyme 1(UFC1), and the E3 ligase complex with UFM1-specific ligase 1 (UFL1) association with the domain-containing protein 1 (DDRGK1) and CDK5 regulatory subunit associated protein 3 (CDK5RAP3). Pharmacological disruption of the DDRGK1–UFL1 interaction with osimertinib or CP-24 reduces global UFMylation, impairs endoplasmic reticulum (ER)-phagy, and increases ER stress. The UFMylation-inhibitory activity and anti-GBM effects of osimertinib were independent of its canonical EGFR-targeting activity. Pharmacological UFMylation inhibition also enhanced the response of GBM cells to TMZ and irradiation and promoted an M1-like antitumor macrophage phenotype, linking this PTM to both tumor-intrinsic resistance and immune regulation (85).

For other emerging PTMs, including mono-ADP-ribosylation, propionylation, butyrylation, malonylation, glutarylation, and hydroxyisobutyrylation, GBM-specific evidence directly linking the modification itself to therapeutic resistance remains insufficient. Further studies are required to determine whether these PTMs participate in treatment response or represent actionable vulnerabilities in GBM.

## 5 PTM crosstalk

PTM crosstalk occurs when one modification influences the installation, removal, recognition, or functional consequence of another, either on the same substrate or through regulatory interactions between PTM-modifying enzymes and their targets (86). Such interdependence can generate functional outputs that are not attributable to individual PTMs alone and may contribute to therapeutic resistance. To help integrate these mechanisms, **Figure 2** summarizes selected examples of PTM crosstalk associated with radiotherapy response and GSC maintenance.

**Figure 2:**
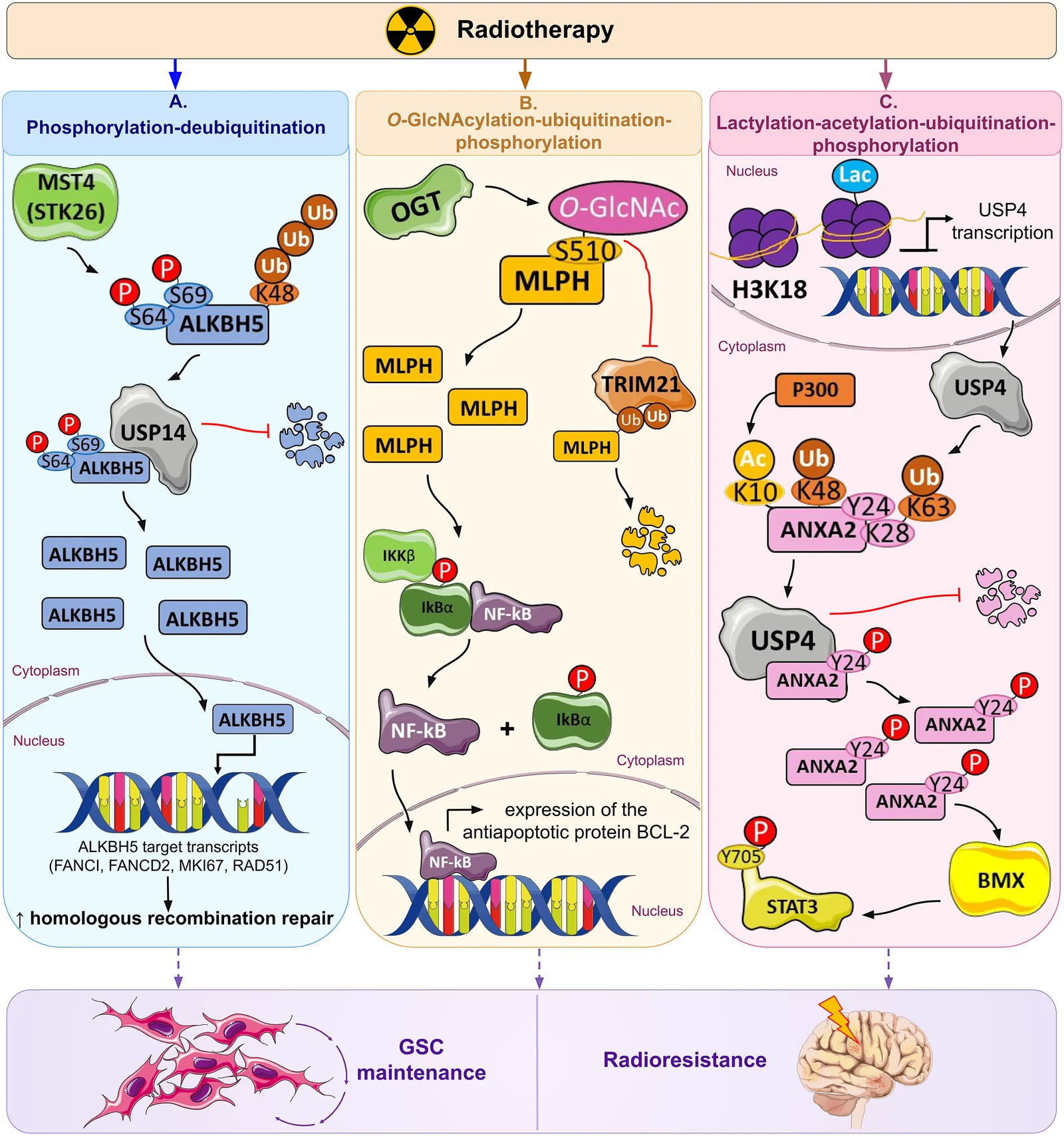
Selected examples of PTM crosstalk in the response to radiotherapy. **(A)** MST4-induced phosphorylation of ALKBH5 at S64/S69, enhances its interaction with USP14, which removes K48-linked ubiquitin chains and prevents ALKBH5 degradation. Increased ALKBH5 levels in GSCs regulate homologous recombination-related ALKBH5 target transcripts and promote GSC maintenance and radioresistance (39). **(B)** OGT-induced O-GlcNAcylation of MLPH at S510, limites TRIM21-mediated ubiquitination and degradation and increases MLPH levels. Increased MLPH activates NF-κB signaling and BCL-2 expression, promoting radioresistance (90). **(C)** H3K18 lactylation increases USP4 expression, while p300 induces ANXA2 acetylation at K10 and enhances its interaction with USP4. USP4 removes K48- and K63-linked ubiquitin chains from ANXA2, and K63-linked ubiquitination at K28 promotes ANXA2 phosphorylation at Y24. These modifications activate the USP4/ANXA2–BMX–STAT3 axis, supporting GSC maintenance and radioresistance (59).

Phosphorylation-dependent control of deubiquitination provides an example of PTM crosstalk in GSCs. Upon exposure to ionizing radiation, mammalian STE20-like protein kinase 4 (MST4) expression increases, accompanied by increased phosphorylation of ALKBH5 at S64 and S69. MST4-mediated phosphorylation enhances ALKBH5 interaction with USP14, promoting USP14-mediated removal of K48-linked ubiquitin chains from ALKBH5, preventing its proteasomal degradation, increasing ALKBH5 levels in GSCs, and promoting radioresistance. Disruption of this phosphorylation–deubiquitination mechanism impairs homologous-recombination repair and increases radiosensitivity (39).

In GSCs, phosphorylation, deubiquitination, and SUMOylation are also functionally connected through the Pin1–Ubc9 axis. Polo-like kinase 1 (PLK1)-dependent phosphorylation of Pin1 at S65 facilitates its interaction with ubiquitin-specific peptidase 34 (USP34), which deubiquitinates and stabilizes Pin1. In parallel, cyclin-dependent kinase 1 (CDK1)-dependent phosphorylation of Ubc9 at S71 promotes its interaction with Pin1. Pin1 then isomerizes phosphorylated Ubc9, enhancing Ubc9–SUMO1 thioester formation and global SUMO1-modified sumoylation. Combined inhibition of Pin1 with sulfopin and CDK1 with RO3306 reduced SUMO1 modification, impaired GSC maintenance, and suppressed intracranial GBM growth, providing pharmacological evidence that this PTM network can be disrupted *in vivo*, although direct effects on TMZ or radiotherapy response were not evaluated (87).

PTM crosstalk also contributes directly to acquired TMZ resistance. In recurrent GBM, reduced phosphorylation of tripartite motif-containing 25 (TRIM25) at S100 impairs its ability to promote K48-linked ubiquitination of inositol-trisphosphate 3-kinase B (ITPKB) at K793 and K818, resulting in ITPKB stabilization. Increased ITPKB supports redox homeostasis and protects resistant cells from TMZ-induced oxidative stress, whereas pharmacological inhibition of ITPKB restores TMZ sensitivity in resistant models (88).

Phosphorylation–methylation crosstalk also contributes to resistance to mTOR inhibition. Following mTOR inhibition, protein kinase A (PKA) phosphorylates PRMT5 at S15, enabling its interaction with hnRNP A1 and subsequent PRMT5-dependent arginine methylation of hnRNP A1 at R218 and R225. This methylation sustains IRES-dependent translation of cyclin D1 and c-Myc despite mTOR blockade. Combined inhibition of PKA and mTOR suppressed the translational program and induced apoptosis *in vitro* and in intracranial xenografts, linking coordinated phosphorylation and methylation to resistance to mTOR inhibition (89).

Crosstalk between O-GlcNAcylation and ubiquitination regulates the response to radiotherapy. Melanophilin (MLPH) was highly expressed in recurrent GBM specimens after radiotherapy, and OGT-mediated *O*-GlcNAcylation of MLPH at S510 affects its interaction with the E3 ligase tripartite motif-containing 21 (TRIM21), limiting TRIM21-mediated ubiquitination and degradation of MLPH. Increased MLPH promotes nuclear NF-κB signaling and expression of the anti-apoptotic protein B-cell lymphoma 2 (BCL-2), contributing to radioresistance. Conversely, pharmacological inhibition of *O*-GlcNAcylation decreases MLPH levels, suppresses NF-κB signaling, and increases GBM radiosensitivity (90).

High USP4 expression is associated with poor prognosis in GBM patients and is preferentially observed in GSCs. H3K18 lactylation promotes transcriptional upregulation of USP4. In parallel, p300 interacts with ANXA2 and regulates its acetylation at K10, enhancing ANXA2 interaction with USP4. USP4 removes K48- and K63-linked ubiquitin chains from ANXA2, preventing its degradation, while K63-linked polyubiquitination of ANXA2 at K28 promotes phosphorylation at Y24. This lactylation–acetylation–ubiquitination–phosphorylation crosstalk converges on the USP4/ANXA2 axis, activating bone marrow kinase X-linked (BMX)-mediated STAT3 signaling and supporting GSC maintenance and radioresistance (59).

Together, these mechanisms suggest that resistance may also emerge from coordinated PTM networks in addition to individual modifications, potentially limiting strategies directed at a single PTM-regulating enzyme.

## 6 Future perspectives and the role of natural compounds

From a clinical perspective, maximal safe resection followed by radiotherapy with concomitant TMZ and followed by maintenance of TMZ remains the treatment backbone for eligible patients with newly diagnosed GBM. At recurrence, systemic treatment is individualized, with options including lomustine, TMZ rechallenge in selected patients, and bevacizumab where available. In parallel, several molecularly targeted approaches have been clinically investigated, including receptor tyrosine kinase inhibitors such as neratinib, dacomitinib, and erlotinib targeting EGFR, MEK inhibition with trametinib, and inhibitors of the PI3K/AKT/mTOR pathway, including everolimus, buparlisib, and paxalisib. Other strategies targeting protein homeostasis, such as proteasome inhibition with marizomib, have also reached clinical evaluation (91).

Interestingly, some of these clinically used or investigational therapies intersect with PTM-dependent mechanisms discussed throughout this review. TMZ and radiotherapy resistance are associated with multiple PTMs and PTM crosstalk. Phosphorylation-dependent signaling has been implicated in adaptation to bevacizumab and targeted inhibition of EGFR and PI3K/AKT/mTOR signaling. Moreover, PRMT5-dependent methylation has been associated with adaptive resistance to the MEK inhibitor trametinib, while deubiquitination has been linked to lomustine response, and PRMT5-dependent methylation and phosphorylation–methylation crosstalk have been implicated in resistance to mTOR inhibition. Together, these examples illustrate how PTM-dependent mechanisms may influence both established treatments and molecularly targeted strategies evaluated in GBM. The main PTM-associated mechanisms linked to therapeutic resistance are summarized in **Table 1**.

**Table 1:** PTM-associated mechanisms linked to therapeutic resistance in glioblastoma.

Resistance	PTM	Model	Refs
TMZ resistance	Phosphorylation/ PI3K/AKT signaling	GBM cell lines, paired pre-/post-treatment GBM, patient-derived, and TMZ-resistant GBM	(15, 27, 28)
	Ubiquitination/deubiquitination	GBM models, including MGMT-proficient and MGMT-deficient cells, patient-derived GBM	(40–44)
	SUMOylation	GBM exposed to genotoxic stress	(48)
	Neddylation	TMZ-resistant GBM cell lines	(51)
	Acetylation/deacetylation	PDX-derived GBM cells, orthotopic PDX, and TMZ-resistant patient-derived GBM	(61, 62)
	Methylation/demethylation	GSC models and treatment-naive and TMZ-exposed patient-derived GBM	(64, 66, 69)
	Phosphorylation + methylation	EGFR-activated GBM	(65)
	Fucosylation	GBM cells	(76)
	*O*-GlcNAcylation	GBM cells, including patient-derived GBM cells	(77, 78)
	Lactylation	Recurrent GBM and TMZ-resistant GBM cells	(82)
Acquired TMZ resistance	Methylation/demethylation	TMZ-resistant GBM cells	(67, 68)
	Phosphorylation + ubiquitination	Recurrent GBM and TMZ-resistant GBM	(88)
TMZ and radiotherapy resistance	Lactylation	ALDH1A3-high GSCs and GBM	(81)
	UFMylation	GBM cells and *in vivo* GBM	(85)
Radioresistance	Lactylation + acetylation + ubiquitination + phosphorylation	GSCs	(59)
	Phosphorylation + deubiquitination	GSCs and GSC-derived brain tumor xenografts	(39)
	Acetylation	GSCs and radiation-treated *in vivo* glioma	(60)
	Sialylation	GSCs and intracranial GSC-derived tumors	(74)
	*O*-GlcNAcylation + ubiquitination	GBM cells, recurrent GBM samples, and xenograft models	(90)
Targeted therapy resistance	Phosphorylation/RTK/MAPK signaling	GBM under EGFR inhibition, patient-derived models, GBM neurospheres; and xenografts under mTOR inhibition	(29–31) (32)
	Phosphorylation	Bevacizumab-resistant patient GBM and GBM models of anti-VEGF resistance	(34)
	Methylation	GBM models under mTOR inhibition; Patient-derived GBM neurospheres and intracranial xenografts under trametinib	(70, 71)
	Phosphorylation + methylation	*In vitro* GBM and intracranial xenograft models under mTOR inhibition	(89)
	Neddylation	GBM with differential response to NAE inhibitors	(52)
Lomustine response	Deubiquitination	GBM cells	(44)

Alongside synthetic drugs aimed at anticancer therapy; natural bioactive compounds have emerged as potential modulators of PTM-dependent pathways relevant to GBM progression and therapeutic resistance. There is evidence that polyphenols, terpenoids, and other natural compounds can influence phosphorylation, ubiquitination, SUMOylation, acetylation, and related signaling networks by modulating PTM-regulating enzymes or pathways that control the modification state of specific proteins (45, 92). Such effects may be relevant to the resistance mechanisms discussed throughout this review. However, most evidence remains preclinical, and changes in PTM-dependent signaling do not necessarily demonstrate direct targeting of the corresponding PTM machinery. Thus, the ability of natural compounds to specifically modulate PTM-dependent resistance mechanisms requires further validation in clinically relevant GBM models.

Work from our group provides complementary evidence that natural compounds and natural-product-derived molecules can affect glioma and GBM. Guanosine exerted cytotoxic effects in A172 glioma cells and potentiated TMZ-induced apoptosis (93). In neural models, the nucleoside guanosine also induced a transient increase in global SUMO2/3 conjugation through an adenosine receptor-dependent mechanism (94), suggesting a potential link between guanosine and SUMOylation that remains to be investigated in GBM. The marine carotenoid fucoxanthin showed antitumor effects in human GBM cells, reducing proliferation, migration, and invasion and increasing apoptosis (95). In addition, the plant lectin ConBr modulated MAPK/AKT signaling and induced autophagy- and caspase-8-dependent cell death in a rat glioma model (96). Among natural-product-derived compounds, the synthetic chalcone Q1VA induced apoptosis and reduced invasion in human GBM cells obtained from surgical resection (97). Together, these findings support the antitumor potential of natural compounds and their derivatives in glioma and GBM, however, whether specific PTMs mediate these effects remains unclear.

Among other natural compounds investigated in glioma, polyphenols have received particular attention. Curcumin, the major bioactive polyphenolic constituent of *Curcuma longa*, has shown antiproliferative effects in preclinical GBM models (92). In patient-derived GSCs, curcumin-induced reactive oxygen species (ROS) altered STAT3 phosphorylation, decreasing phosphorylation at Y705 while increasing phosphorylation at S727. These changes were accompanied by reduced STAT3-dependent signaling, proliferation, and self-renewal (98).

The stilbene polyphenol resveratrol and the diterpenoid quinone cryptotanshinone further link STAT3 phosphorylation to TMZ response. Combined resveratrol and TMZ treatment enhanced TMZ response and reversed resistance in LN428 GBM cells, with reduced STAT3 activity and MGMT expression; the JAK2-specific inhibitor AG490 similarly reduced MGMT through STAT3 inactivation (99). Likewise, cryptotanshinone inhibits STAT3 Y705 phosphorylation and, when combined with TMZ, reduces MGMT expression, increases DNA damage, and restores TMZ responsiveness in acquired TMZ-resistant U251 and U87 models (100). Phosphorylation-dependent effects on resistant GBM cells have also been demonstrated for thymoquinone, a bioactive monoterpene quinone derived from *Nigella sativa*. In TMZ-resistant U251R cells, thymoquinone increases p38 MAPK phosphorylation and induces apoptosis. Pharmacological inhibition or silencing of p38 attenuates this effect, indicating that p38 activation is required for the response of TMZ-resistant cells to thymoquinone (101).

Ubiquitin-dependent regulation directly links natural-compound activity to TMZ response. In TMZ-resistant T98G-R and GBM3-R cells, Sanggenol L, a mulberry-derived flavonoid, promotes K48-linked ubiquitination and degradation of MGMT, with lysine 18 (K18) identified as the predominant site involved in Sanggenol L-induced MGMT ubiquitination. This effect is associated with reduced USP7 levels and enhanced TMZ-induced DNA damage and apoptosis in resistant models (102). Additionally, Sanggenol L increases tripartite motif-containing 16 (TRIM16) expression, promoting ubiquitination and proteasomal degradation of the mitophagy receptor optineurin (OPTN), disrupting protective mitophagy and enhancing TMZ sensitivity (103).

Natural compounds can also influence ubiquitin-like modifications. Chlorogenic acid increases SUMO1 expression and promotes c-Myc SUMOylation, miR-17-family suppression, and p21 induction in Huh7 and H446 cancer models. In U87MG and M059J glioma cells, chlorogenic acid reduced p-c-Myc, increased p21 expression, and induced differentiation-associated changes (104). Chlorogenic acid is among the few natural compounds discussed here to have progressed to clinical evaluation in glioma. Although SUMOylation was not evaluated as a pharmacodynamic endpoint, a phase I dose-escalation study in recurrent high-grade glioma showed that injectable chlorogenic acid was well tolerated and resulted in stable disease in 12 of 23 evaluable patients after the first treatment cycle; median overall survival was 11.3 months (105).

Berberine is a plant-derived isoquinoline alkaloid that has been investigated for its antitumor activity in GBM. In GSCs, pharmacological inhibition of MCU with berberine reduced the activity of an MCU-dependent metabolic–acetylation axis in which mitochondrial Ca²^+^ uptake increases acetyl-CoA availability and H3K27 acetylation at the tribbles pseudokinase 3 (*TRIB3*) locus. Berberine attenuated H3K27 acetylation and TRIB3 expression, suppressed GSC growth, and prolonged survival in mouse GBM models (58).

Given the marked heterogeneity of GBM, future studies should define which PTM-dependent resistance mechanisms are most amenable to modulation by natural compounds and identify the tumor contexts in which such interventions are most effective. Natural compounds can therefore affect several PTM-regulated processes, with direct evidence for modulation of therapeutic resistance for phosphorylation-dependent signaling and ubiquitin-mediated protein turnover. A major challenge is to distinguish causal modulation of PTM-dependent resistance from secondary changes accompanying compound-induced cytotoxicity. Integrating PTM profiling with treatment-response studies in patient-derived and *in vivo* models may help define tumor contexts associated with treatment response and support rational combinations of natural compounds with existing GBM therapies.

## 7 Conclusion

PTMs contribute to GBM therapy resistance by regulating signaling, protein stability, DNA repair, metabolism, and stem-cell-associated mechanisms. Recent studies have identified specific PTM-dependent vulnerabilities that can modify responses to TMZ, radiotherapy, and targeted therapies, but most evidence remains preclinical and strongly dependent on molecular context. Further validation in patient-derived and *in vivo* models will be necessary to determine which PTM-regulated mechanisms can be translated into effective therapeutic strategies.

## Glossary

α-KG: α-ketoglutarate
ADP: adenosine-5'-diphosphate
AKT: protein kinase B
ALDH1A1: aldehyde dehydrogenase 1 family member A1
ALKBH2: AlkB homolog 2
ALKBH5: AlkB homolog 5
ANXA2: annexin A2
AP-1: activator protein 1
ATG9B: autophagy-related 9B
BCAT1: branched-chain amino acid transaminase 1
BCL-2: B-cell lymphoma 2
BMX: bone marrow kinase X-linked
BRCA1: breast cancer type 1 susceptibility protein
CCNU-Lomustine: chloroethyl-cyclohexyl-nitrosourea
CDK1: cyclin-dependent kinase 1
CDK5RAP3: CDK5 regulatory subunit associated protein 3
CHIP: C-terminus of Hsc70-interacting protein
CNS: central nervous system
CoA: coenzyme A
COP9: constitutive photomorphogenesis 9
CSN5: COP9 signalosome homolog subunit 5
DCA: dichloroacetate
DDR: DNA damage response
DDRGK1: DDRGK domain-containing protein 1
DNA: deoxyribonucleic acid
DUB: deubiquitinase
E2F6: E2 transcription fator 6
ECM: extracelular matrix
EGFR: epidermal growth factor receptor
EGFR-TKI: EGFR tyrosine kinase inhibitor
EGFRvIII: EGFR variant III
ELK1: ETS-like protein 1
ER: endoplasmic reticulum
ERBB3: receptor tyrosine-protein kinase erbB-3
ERK: extracellular signal-regulated kinase
FA: Fanconi anemia
FAK: focal adhesion kinase
FGFR: fibroblast growth factor receptor
FUT8: fucosyltransferase 8
GBM: glioblastoma
GDH1: glutamate**;** GDH1, dehydrogenase 1 gene and protein
GLI1: glioma-associated oncogene family zinc finger 1
GSC: glioblastoma stem cells
H2AX: histone H2A.X
H3K9: histone H3 lysine 9
H3K 18: histone H3 lysine 18
H3K 27: histone H3 lysine 27
HAT: histone acetyltransferase
HDAC: histone deacetylase
HERC5: HECT and RLD domain containing E3 ubiquitin protein ligase 5
HGF: hepatocyte growth factor
HIF-1α: HIF-2α, hypoxia-inducible factor 1 alpha or 2 alpha
HK2: hexoquinase 2
HMOX1: heme oxygenase 1 gene
hnRNP: heterogeneous nuclear ribonucleoprotein
HO-1: heme oxygenase 1 protein
IDH: isocitrate dehydrogenase
IGF1R: insulin-like growth factor 1 receptor
IL-10: interleukin-10
InsR: insulin receptor
IRES: internal ribosome entry site
ISG15: interferon-stimulated gene 15
ITPKB: inositol-triphosphate 3-kinase B
JAK: Janus kinase
JAK2: Janus kinase 2
JNK: c-Jun N-terminal kinase
KAT: lysine acetyltransferase
KDM or KDM6A: lysine demethylase or lysine demethylase 6A
Kme1: Kme2, Kme3, mono-. di- and tri-methylated lysine
KMT: lysine methyltransferase
KRT8: keratin 8
LMO7: LIM domain only 7
LUC7L2: RNA-binding protein LUC7-like 2
MAPK: mitogen-activated protein kinase
MCU: mitochondrial calcium uniporter
MDC1: mediator of DNA damage checkpoint 1
MEK: mitogen-activated protein kinase kinase
MET: hepatocyte growth factor receptor
MGMT: O6-methylguanine-DNA methyltransferase
miR-17, 204-3p, or 616: microRNA 17, microRNA 204-3p, microRNA 616
MLH1: MutL homolog 1
MLPH: melanophilin
MST4: mammalian STE20-like protein kinase 4
MTA: 5′-deoxy-5′-methylthioadenosine
MTAP: methyladenosine phosphorylase
mTOR: mechanistic target of rapamycin
NAE: NEDD8-activating enzyme
NANP: N-acylneuraminate-9-phosphatase
NEDD8: neural precursor cell expressed developmentally downregulated 8
NF-κB: nuclear factor kappa B
NFAT5: nuclear factor of activated T cells 5
Oct4: octamer-binding transcription factor 4
OGA: O-GlcNAcase
O-GlcNAc: O-linked β-N-acetylglucosamine
OGT: O-GlcNAc transferase
Olig2: oligodentrocyte transcription factor 2
OPTN: optineurin
PCNA: proliferating cell nuclear antigen
PDGFB: platelet-derived growth factor subunit B
PDGFRβ: platelet-derived growth factor receptor β
PDK1: pyruvate dehydrogenase kinase 1
PDX: patient-derived xenograft
PGK1: phosphoglycerate kinase 1
PI3K: phosphoinositide 3-kinase
PIAS: protein inhibitor of activated STAT
Pin1: peptidyl-prolyl cis-trans isomerase NIMA-interacting 1
PKA: protein kinase A
PLK1: polo-like kinase 1
PML: promyelocytic leukemia protein
Polκ: DNA polymerase kappa
PRMT5: protein arginine methyltransferase 5
PTEN: phosphatase and tensin homolog
PTM: post-translational modifications
RAD51: radiation sensitive protein 51
RBBP4: retinoblastoma-binding protein 4
RBX1: RING-box protein 1
RBX2: RING-box protein 2
RING: really interesting new gene finger domain
ROS: reactive oxygen species
RTK: receptor tyrosine kinase
SAM: S-adenosylmethionine
SENP: Sentrin-specific proteases
Siglec: sialic acid-binding immunoglobulin-like lectin
SOX2: SRY-box transcription factor 2
SREBP: sterol response element-binding protein
STAT3: signal transducer and activator of transcription 3
SUMO: small ubiquitin-like modifier
SUV39H1: suppressor of variegation 3-9 homolog 1
TAM: tumor-associated macrophage
TCA: tricarboxylic acid
TF: transcription factor
TME: tumor microenvironment
TMZ: temozolomide
TNF: tumor necrosis factor
TNFR1: tumor necrosis factor receptor 1
TP53BP1: tumor protein p53-binding protein 1
TRIB3 TRIB3: tribbles pseudokinase 3 gene and protein
TRIM16: TRIM21, TRIM25 , tripartite motif-containing 16, 21 or 25
UBA3, 5 or 7: ubiquitin-associated domain 3, 5 or 7
Ubc9 UbcH8: ubiquitin-conjugating enzyme 9 or H8
UBE2F UBE2M: ubiquitin conjugating enzyme E2 F or M
UBL: ubiquitin-like protein
UCHL1: uniquitin C-terminal hydrolase L1
UFC1: ubiquitin-fold modifier conjugating enzyme 1
UFL1: UFM1-specific ligase 1
UFM1: ubiquitin-fold modifier 1
USP7: USP 14, USP 34, ubiquitin-specific peptidase 7, 14 or 34
VC-Resist: vessel-co-opting GBM
VEGF: vascular endothelial growth factor
VEGFR: vascular endothelial growth factor receptor
VM: vasculogenic mimicry
XRCC1: X-ray repair cross-complementing protein 1
